# Lipid-lowering therapy (LLT) in 1,100 cardiac rehabilitation patients with coronary heart disease: the LLT-R(ehabilitation) registry

**DOI:** 10.3389/fcvm.2025.1549935

**Published:** 2025-04-24

**Authors:** Frank Noack, Kristina Eckrich, Heinz Völler, Bernhard Schwaab, Viktoria Heinze, Christa Bongarth, Manju Guha, Michael Richter, Nadja Schwark, Alexandra Strobel, Axel Schlitt

**Affiliations:** ^1^ Department of Emergency Medicine, University Clinic Halle-Saale, Halle, Germany; ^2^ Medical Faculty, Martin Luther-University Halle Wittenberg, Halle, Germany; ^3^ Department of Cardiology, Clinic Tharandter Wald-Hetzdorf, Halsbrücke, Germany; ^4^ Department of Cardiology, Klinik am See, Rüdersdorf, Germany; ^5^ Common Faculty of the Brandenburg University of Technology Cottbus – Senftenberg, Cottbus, Germany; ^6^ The Brandenburg Medical School, Theodor Fontane and the University of Potsdam, Potsdam, Germany; ^7^ Curschmann-Clinic Timmendorfer Strand and Medical Faculty, University of Lübeck, Lübeck, Germany; ^8^ Department of Cardiology, Paracelsus-Harz-Klinik Bad Suderode, Quedlinburg, Germany; ^9^ Department of Cardiology, Clinic Höhenried, Bernried, Germany; ^10^ Department of Cardiology, Rehabilitation Clinic Sendesaal, Bremen, Germany; ^11^ Coordination Center for Clinical Studies, Martin Luther-University Halle Wittenberg, Halle, Germany; ^12^ Institute of Medical Epidemiology, Biostatistics, and Informatics, Martin Luther-University Halle, Halle, Germany

**Keywords:** lipid-lowering therapy, cardiac rehabilitation, lipids, coronary heart disease, statins

## Abstract

**Background:**

Cardiac rehabilitation (CR) improves quality of life and prognosis in patients with coronary heart disease (CHD). The aim of the study was to evaluate effects of lipid-lowering therapy (LLT) in patients with CHD during and after CR.

**Methods:**

Data from prospective, multicenter registry including 1,100 patients with CHD undergoing CR in 6 German cardiac rehabilitation centers between 2016 and 2018 were analyzed.

**Results:**

The rate of statin-treated patients increased from 1,048 (96.3%) on admission to 1,062 (98.4%) at discharge (*p* < 0.001), falling to 644 (96.3%) and 609 (94.1%) at 3 and 12 months, respectively. Combination treatment with ezetimibe was effective in 8.9% of patients at admission and 28.5% at discharge (*p* < 0.001), and 23.5% and 25.8% after 3 month and 12 months, respectively. Titration of LLT during CR resulted in median LDL-C-values of 2.27 mmol/L at admission, 1.97 mmol/L at discharge (*p* < 0.001), 1.94 mmol/L after 3 months, and 1.94 mmol/L after 12 months, respectively.

**Conclusions:**

During CR, LLT was effectively instituted and titrated, resulting in a high rate of statin-treated patients and a significant reduction in LDL-C. From this study, we hypothesize that CR is efficacious for adherence to LLT.

## Background

Coronary atherosclerosis and its clinical manifestation of coronary heart disease (CHD) is the leading cause of death worldwide. As CHD is an age-related disease and life expectancy is increasing, especially in western countries, the number of patients with CHD is also increasing. Thus, preventing CHD by treating risk factors such as dyslipidemia, hypertension, or diabetes represents a challenge for health systems worldwide, too (1).

After a diagnosis of CHD, treatment includes life-style changes such as a healthier diet, regular exercise, and smoking cessation. Evidence supports medical therapies such as platelet inhibitors, ACE inhibitors (ACE-I) and angiotensin-receptor blockers (ARB), beta-blocking agents, and, particularly, lipid-lowering drugs in improving mortality in CHD patients (1, 2). Furthermore, patients with established CHD should be treated with lipid-lowering therapy (LLT), irrespective of the initial low-density lipoprotein cholesterol (LDL-C) levels. New goals of treatment in patients with CHD who are considered to be at very high risk are LDL-C levels below 1.4 mmol/L (55 mg/dl) and an LDL-C reduction of ≥50% from baseline in LDL-C, irrespective of starting LDL-C (2). To reach these goals, statin treatment is recommended, up to the highest tolerated dose. In some patients, however, treatment targets cannot be achieved by statin monotherapy. The evidence for fibrate treatment is weak cholesteryl ester transfer protein (CETP) inhibitors are not available, and niacin has failed to reduce cardiovascular events (3); thus, additional treatment options for those individuals who do not respond adequately or who do not tolerate statin therapy include combination therapy with ezetimibe and proprotein convertase subtilisin/kexin type 9 (PCSK9) inhibitors, which have been shown to be effective in reducing LDL-C (2–4). The addition of ezetimibe to simvastatin therapy resulted in a modest absolute reduction (1.8%) of a combined endpoint consisting of death, nonfatal myocardial infarction, or non-fatal/over a seven year follow-up period (4). Alirocumab and evolocumab (both PCSK9 inhibitors) reduced LDL-C by approximately 50% in patients who were treated with high-dose statins (with or without ezetimibe) and have demonstrated improvements in major clinical endpoints in recent trials (5, 6). Although LLT generally is well tolerated and efficacious in improving the life expectancy of patients with CHD, problems do arise in adherence to therapy (7).

To reduce the high risk of dying after an acute coronary syndrome (ACS) the implementation of guideline-orientated target values is important, which can be achieved with a multicomponent cardiac rehabilitation (1). According to a recent meta-analysis, CR participation is effective in reducing mortality in patients after ACS, after coronary artery bypass surgery (CABG) or in mixed coronary artery disease populations (8). Such CR programs differ in length and content in different countries, however (8, 9). In Germany, after hospitalization for ACS or after CABG, at least 50% of patients were transferred to one of more than 150 CR clinics within 14 days after discharge (10, 11). The mean length of stay in rehabilitation clinics is approximately 3 weeks, during which medical therapy is optimized, as treatment targets have often not yet been achieved at the point of admission to the rehabilitation clinics (10, 11). Depending on physical fitness, severity of primary disease, comorbidities, and other confounders, the sports and rehabilitation program is generally set up the day after admission. Patients in good clinical condition participate e.g., every day (except Sunday) in heart rate-monitored 30-minute cycle ergometry training. Moreover, these patients can participate in Nordic walking, medical training therapy, intensive gymnastics/exercise, and functional exercise as aquatic therapy. Patients who are less physically fit can participate in group exercises, chair exercising, and walking exercises, and personal training sessions. All patients, independent of their physical fitness, receive attended seminars and lectures as part of the 3-week rehabilitation program. The costs for such residential rehabilitation programs is borne by either the pension agency insurers or the health insurance agencies in Germany. All of the costs (medical, residential and meal costs) are paid by the allocated insurer.

The aim of this multicenter registry in a rehabilitation setting was to provide information on how patients are treated with LLT compared to lipid therapy targets during and after CR.

## Methods

### Study design

In all, 1,100 patients admitted for CR to the six participating German rehabilitation clinics and in whom LLT was indicated were included. Other inclusion criteria were an age over 18 years, CHD, and admission to one of the participating rehabilitation clinics. Exclusion criteria were inability to give written informed consent or to participate in the registry. The Coordination Center for Clinical Studies, Martin Luther-University Halle Wittenberg, Germany (KKS Halle) developed a monitoring protocol for the study. The local ethics committee of the Medical Association of Saxony-Anhalt approved the study protocol. The ClinicalTrials.gov Identifier was NCT02749279.

### Data collection

All relevant baseline parameters (indication for rehabilitation, LLT, and other drug treatment, all clinical diagnoses, age, sex, BMI, echocardiographic parameters such as left ventricular ejection fraction, laboratory parameters corresponding to the recording standard of the clinic, including LDL-cholesterol and other lipid parameters, etc.) were recorded in a central database (online-CRF). Furthermore, as one of the main topics of interest of the study, LLT at baseline, during the rehabilitation phase, and at discharge and advice given to general practitioners regarding LLT after discharge were collected. After discharge, patients were contacted by mail after 3 and 12 months. Here, general questions about drug therapy, rehospitalizations (particularly in connection with atherosclerotic diseases such as recurrent ACS, and others) were recorded. Drug therapy (particularly LLT) was documented on discharge and at 3- and 12-month follow-up. Data were also collected on the rationale for changes in medication and on who initiated the change. Furthermore, data were collected on lipid markers such as LDL-C, HDL-C, total cholesterol, and triglycerides. Patients who did not send back the questionnaires were contacted by telephone and an interview was conducted with the patient or his/her relatives. In some cases, the patient's physician was contacted. If the information could not be obtained from these sources, civil registration offices were contacted and information was requested about current addresses or date of death.

### Statistical analyses

Continuous variables were described as mean and standard deviation or in case of skewed variables median and minimum/maximum. Categorical variables were documented as absolute numbers and percentages. To compare metric outcomes over time, a repeated measure ANOVA was conducted. Additionally, Cochran's *Q*-Test was conducted to analyze variation of proportions over time. The significance level was set to 5%, however results are all of explanatory nature. Importantly, *p*-values were not adjusted for multiple testing. Missing data was excluded if present and relevant for a statistical analysis. Statistical analyses were performed using SPSS Statistics (IBM® SPSS® Statistics 25, Chicago, IL) software.

## Results

### Patient characteristics

Patient characteristics (age, gender, diagnosis, smoking status, and medication) routinely collected during a CR setting are displayed in Table 1. The average age of the patients was 63.4 ± 10.4 years, the mean BMI 28.6 ± 4.7 kg/m^2^, and 23.6% (262/1,100) of the patients were female. Furthermore, 12.2% (130/1,069) were active smokers, 91.6% (998/1,090) reported dyslipidemia, 33.9% (370/1,090) had diabetes mellitus, and 86.5% (943/1,090) suffered from hypertension. Vital parameters during the study period are shown in Table 2. The index event before entering CR was non-ST-segment elevation myocardial infarction (NSTEMI) in 31.6% (345/1,092) of patients, ST-segment elevation myocardial infarction (STEMI) in 29.6% (323/1,092), and CABG surgery in 26.4% (288/1,092). A follow-up was obtained in 86.9% of patients.

**Table 1:** Patient characteristics (*n* = 1,100).

Variable		
Age (years)	63.4 ± 10.4	
Gender		
Female	262 (23.6%)	
Male	838 (76.4%)	
Diabetes mellitus, *n* = 1,090	370 (33.9%)	
Dyslipoproteinemie, *n* = 1,090	998 (91.6%)	
PAD, *n* = 1,090	77 (7.1%)	
LVEF, *n* = 751	53.9% ± 10.2%	
Arterielle Hypertension, *n* = 1,090	943 (86.5%)	
Smoking, *n* = 1,069		
Never	348 (32.5%)	
Current	130 (12.2%)	
Previous	591 (55.3%)	
Drugs	Admission	Discharge
Platelet inhibitors	98.1% (1,067/1,088)	96.5% (1,040/1,078)
Aspirin	97.5% (1,032/1,059)	96.1% (988/1,028)
Clopidogrel	25.8% (252/975)	27.7% (262/941)
Prasugrel	23.2% (224/649)	24% (223/931)
Ticagrelor	34.4% (343/998)	34.3% (331/964)
Other	0.2% (2/995)	0.2% (2/919)
Oral Anticoagulants	14.1% (155/1,088)	15.3% (165/1,077)
Vitamin-K-Antagonist	33.3% (66/195)	34.2% (69/202)
Dabigatan	27.5% (52/190)	2.2% (4/186)
Rivaroxaban	2.8% (5/181)	30.6% (60/904)
Edoxaban	3.3% (6/181)	2.7% (5/187)
Apixaban	14.0% (26/186)	14.9% (28/189)
Antidiabetics	25.6% (278/1,088)	25.7% (276/1,078)
Metformin	53.3% (181/338)	61.1% (201/329)
Sulfonylurea	6.0% (19/320)	2.3% (7/311)
DPP-4-Inhibitors	30.6% (99/222)	34.5% (108/313)
GLP-1-Agonists	2.5% 8/321)	2.9% (9/312)
SGTL-2-Inhibitors	4.4% (14/320)	7.3% (23/313)
Glinide	0.6% 2/319)	0.6% (2/312)
Insulin	38.1% (124/325)	34.9% (110/315)
Other	1.6% (5/323)	2.5% (8/317)
Antihypertensives	99.4 (1,079/1,088)	99.7 (1,084/1,088)
Diuretics	44.5% (449/1,009)	42.2% (414/981)
ACE-Inhibitors	63.3% (658/1,040)	56.1% (572/1,020)
ARB	31.7% (308/974)	36.2% (345/954)
Renin inhibitors	0.3% (3/970)	0.6% (6/946)
Ca-Antagonists	22.2% (221/989)	24.3% (233/959)
Beta-Blocker	90.3% (964/1,067)	90.6% (955/1,054)
MRA	12.5% (121/972)	10.2% (97/950)

**Table 2:** Vital parameters during the study period (*n* = 1,100).

Variables	Admission	Demission	3-month follow-up	12-month follow-up
BMI (kg/m²)	28.6 ± 4.7	28.6 ± 4.6	28.2 ± 4.3	28.3 ± 4.3
Waist circumference (cm)	103.5 ± 12.7	102.4 ± 12.7	103.9 ± 11.7	103.4 ± 11.6
Systolic blood pressure (mmHg)	134 ± 21.2	130.0 ± 17.5	127.0 ± 12.6	127.0 ± 12.2
Diastolic blood pressure (mmHg)	78.1 ± 11.5	77.7 ± 29.8	76.0 ± 8.9	76.0 ± 10.1
Heart rate (bpm)	73.7 ± 12.4	71.2 ± 11.3	67.0 ± 9.5	67.0 ± 10.0

### LLT during study period

**Figure 1:**
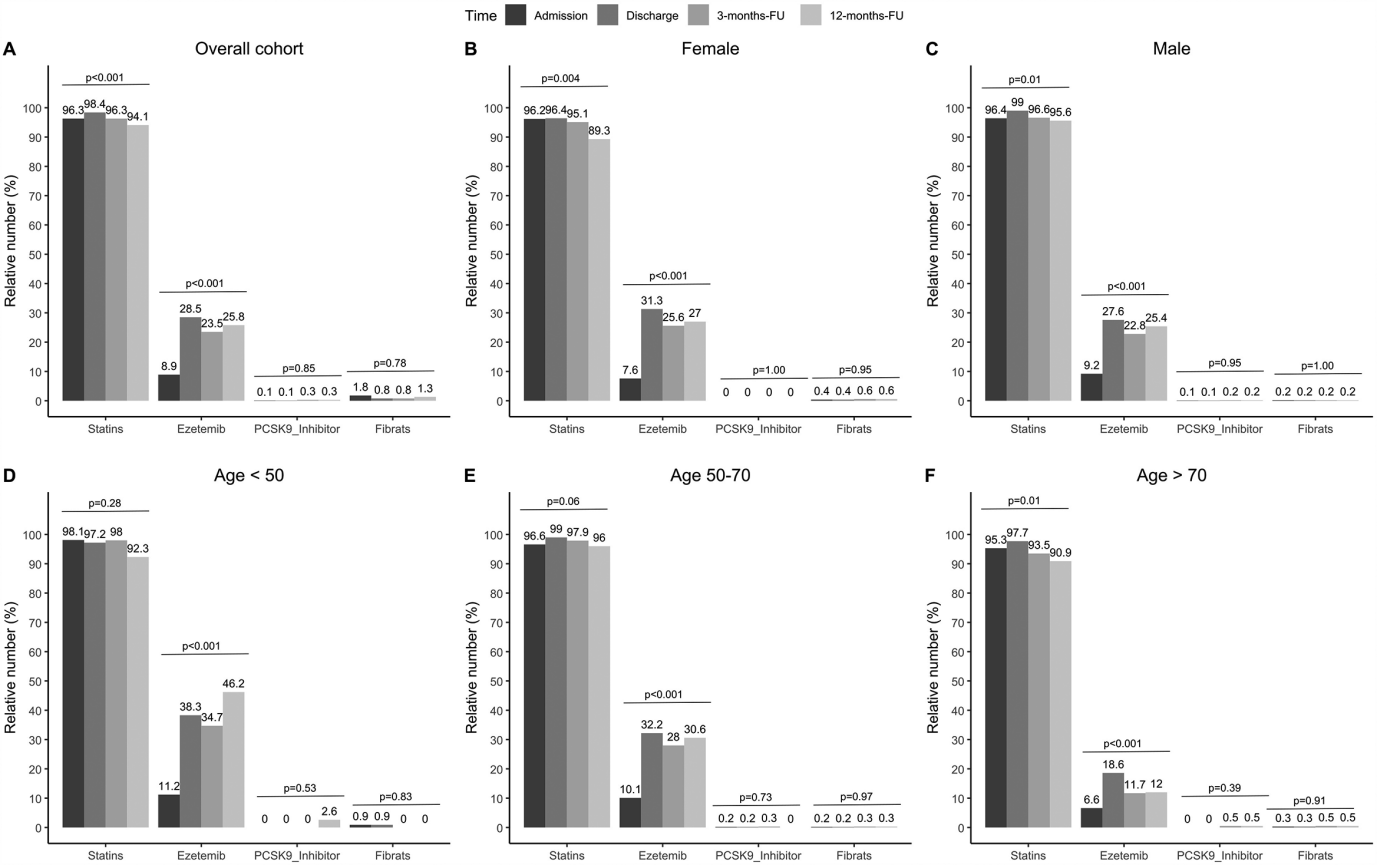
Relative numbers of LLT for admission, discharge and two follow-ups. Results are shown for overall cohort (**A**, *n* = 1,100), stratified by sex (**B,C**, female: *n* = 262, male: *n* = 838), and age groups at baseline (**D–F**, age <50: *n* = 108, age 50–70: *n* = 622, age >70: *n* = 321). *P*-values show results from Cochran's *Q*-Test, comparing proportions over time.

**Figure 2:**
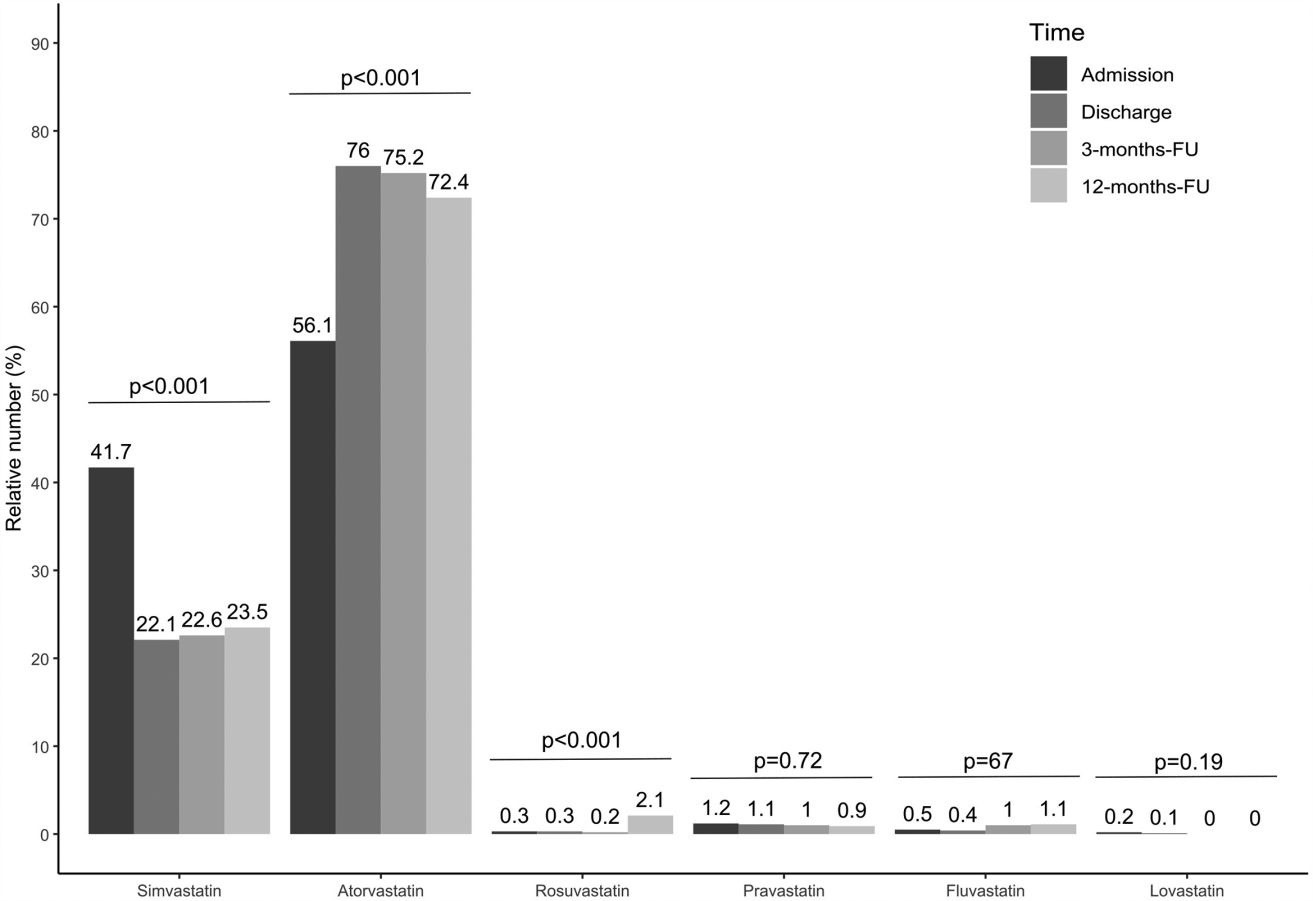
Relative number of statin treatment for admission, discharge and two follow-ups. Results are shown for overall cohort. Valid data (i.e., non-missing values) was available for *n* = 980 patients at admission, *n* = 943 patients at discharge, *n* = 609 at 3-months-FU and *n* = 531 at 12-months-FU. *P*-values show results from Cochran's *Q*-Test, comparing proportions over time.

**Figure 3:**
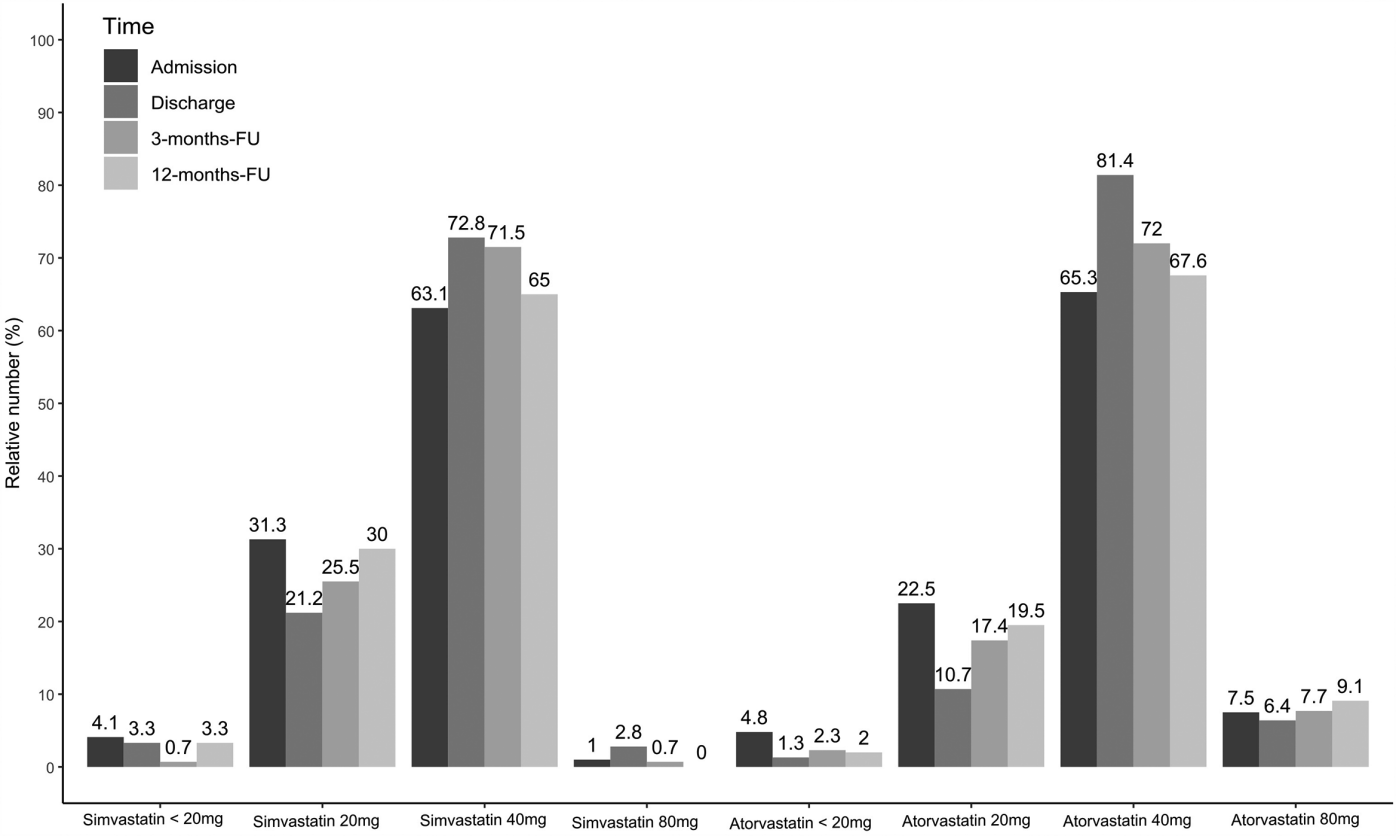
Simvastatin and atorvastatin dosages for admission, discharge and two follow-ups. Results are shown for overall cohort. Valid data (i.e., non-missing values) was available for *n* = 980 patients at admission, *n* = 943 patients at discharge, *n* = 609 at 3-months-FU and *n* = 531 at 12-months-FU.

The proportion of statin-treated patients increased from 96.3% (1,048/1,088) at admission to 98.4% (1,062/1,079) at discharge, falling to 96.3% (644/669) after three and 94.1% (609/647) at 12 months (see Figure 1A, *p* < 0.001). Ezetimibe was part of the therapy in 8.9% (97/1,088) of patients on admission, 28.5% (308/1,081) at discharge, 23.5% (157/669) at 3 months, and 25.8% (167/647) at 12 months (Figure 1A, *p* < 0.001). PCSK9 inhibitors were used in only a rare number of patients without relevant variation over time (Figure 1A, *p* = 0.85). Moreover, no age- or sex-differences can be observed regarding LLT (Figures 1B–F), and the variation over time in these subgroups equal that from the overall cohort. We observed that low-potency statins (such as simvastatin) were replaced by high-potency statins (such as atorvastatin) in a substantial proportion of patients during CR and this drug treatment was maintained through the follow-up period (Figure 2, *p* < 0.001). There was also an increase in the statin dose during CR. During follow-up, however, doses were reduced by one year after CR (Figure 3).

### LDL-C levels during study period

**Figure 4:**
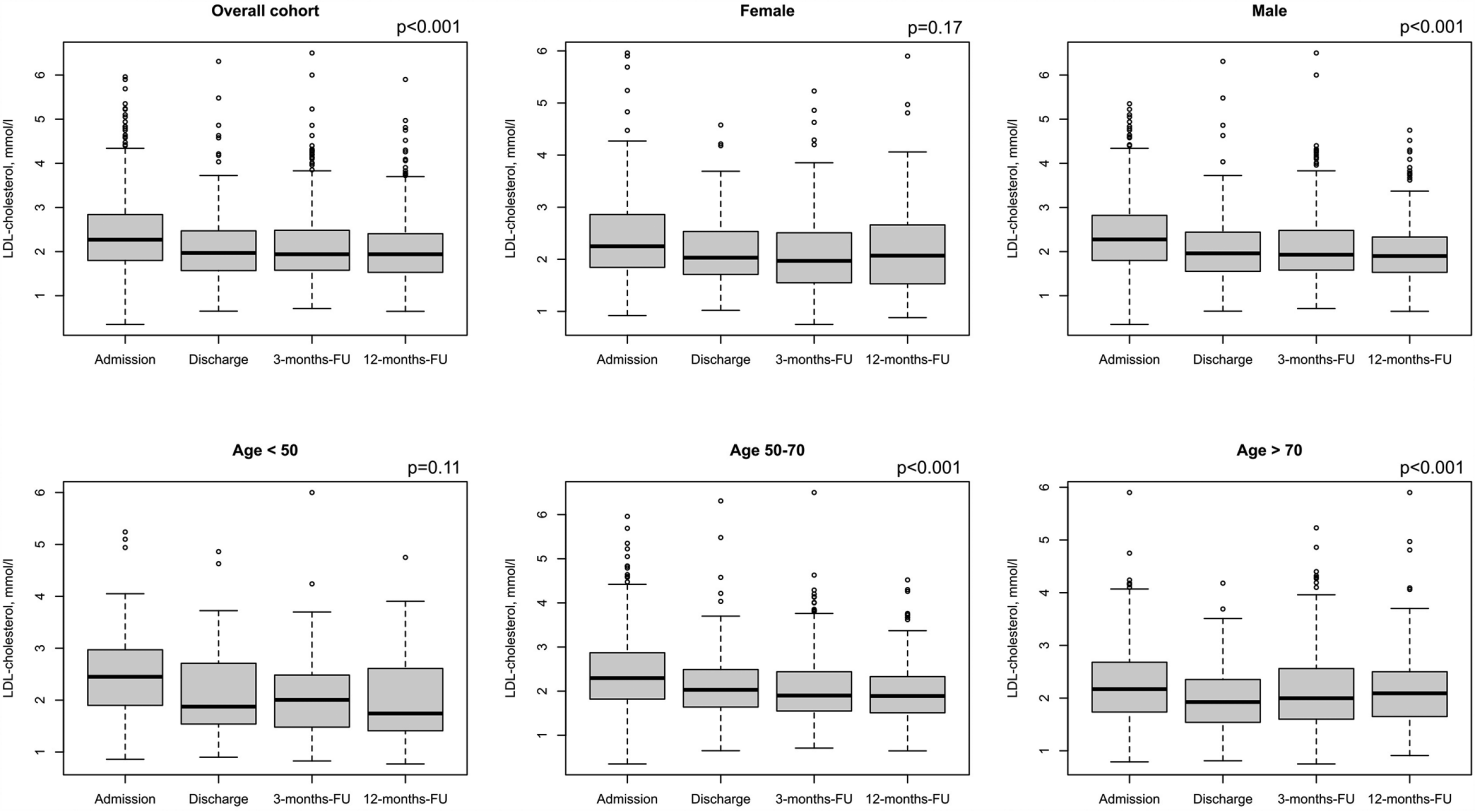
LDL-C values for admission, discharge and two follow-ups. Results are shown for overall cohort (**A**, *n* = 1,100), stratified by sex (**B,C**, female: *n* = 262, male: *n* = 838), and age groups at baseline (**D–F**, age <50: *n* = 108, age 50–70: *n* = 622, age >70: *n* = 321). Regarding LDL-C, valid data (i.e., non-missing values) was available for *n* = 1,089 patients at admission, *n* = 363 patients at discharge, *n* = 533 at 3-months Fu and *n* = 523 at 12-months-FU. *P*-values show results from repeated measures ANOVA.

**Figure 5:**
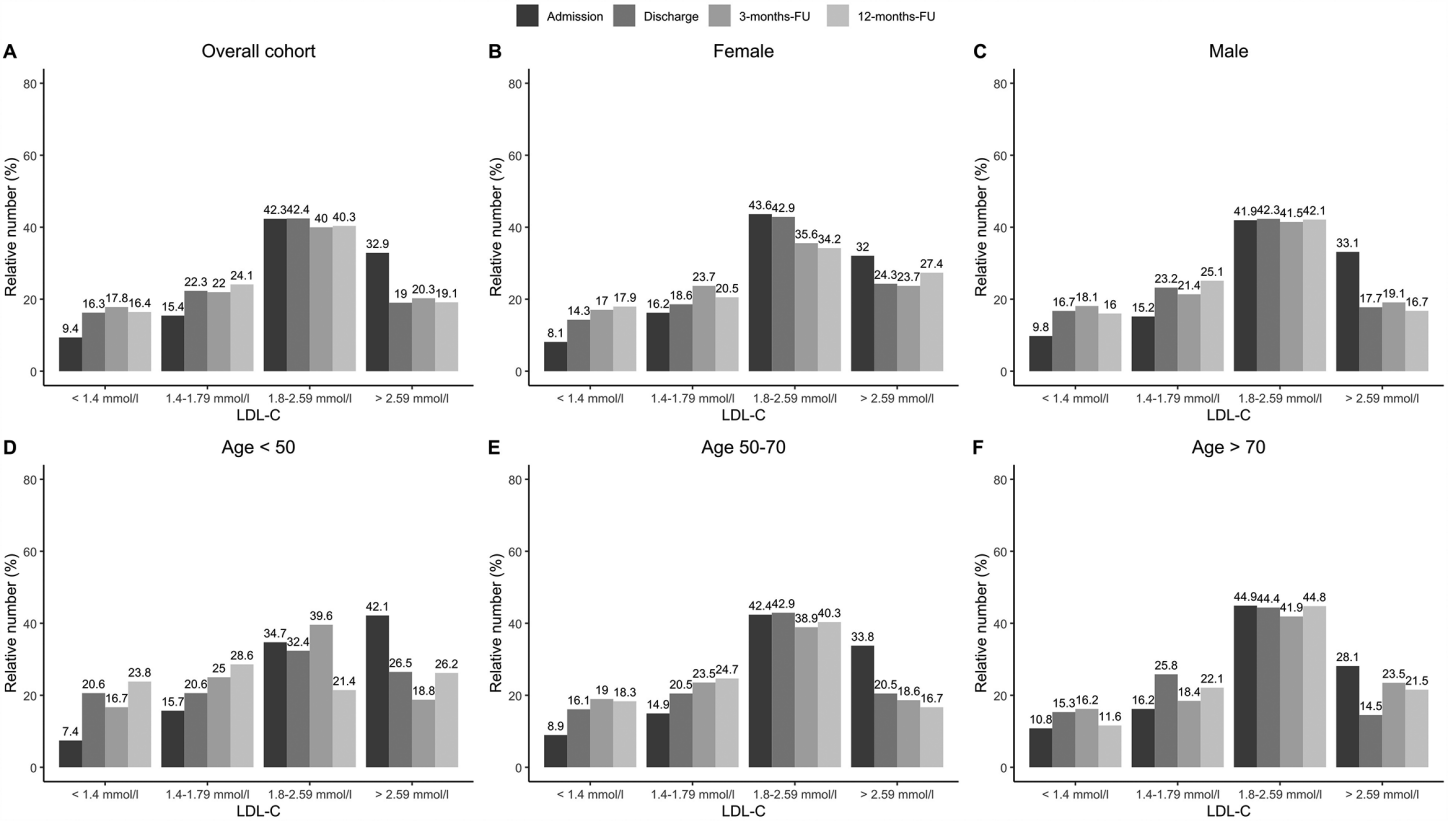
LDL-C ranges achieved at admission, discharge and two follow-ups. Results are shown for overall cohort (**A**, *n* = 1,100), stratified by sex (**B,C**, female: *n* = 262, male: *n* = 838), and age groups at baseline (**D–F**, age <50: *n* = 108, age 50–70: *n* = 622, age >70: *n* = 321). Regarding LDL-C, valid data (i.e., non-missing values) was available for *n* = 1,089 patients at admission, *n* = 363 patients at discharge, *n* = 533 at 3-months Fu and *n* = 523 at 12-months-FU.

Treatment during CR, including adjustment of LLT, nutritional therapy, exercise etc, resulted in median LDL-C levels of 2.27 mmol/L (1.80/2.84, 88.4 mg/dl) at admission and 1.97 mmol/L (1.57/2.47, 76.7 mg/dl) at discharge, showing a relative reduction of 13.2%. In addition, a median LDL-C level of 1.94 mmol/L (1.57/2.49, 75.5 mg/dl) at 3 months, and 1.94 mmol/L (1.53/2.40, 75.5 mg/dl) at 12 months was achieved (Figure 4A). This observed reduction over time was statistically significant (*p* < 0.001) for the overall cohort. However, stratifying results by sex and age showed that male patients achieved a change in LDL-C values from admission to 12-months follow up of 0.35 mmol/L (Figure 4C, *p* < 0.001) over time, while female patients reduced the LDL-C values by 0.21 mmol/L (Figure 4B, *p* = 0.11). In addition, LDL-C values of middle-aged patients continuously decreased from 2.39 mmol/L at admission to 1.98 mmol/L over time (Figure 4E, *p* < 0.001). Similar results were observed from older patients (Figure 4F, *p* < 0.001). Furthermore, we compared at baseline atorvastatin 40–80 mg as monotherapy in the first strata, the combinations of simvastatin (10–40 mg) and atorvastatin 10–20 mg in combination with ezetemibe in the second strata, atorvastatin 40–80 mg in combination with ezetemibe in the third strata, and all other patients in the fourth strata. Afterwards, we analyzed LDL-C levels at admission and found LDL-C levels of 2.22 (±0.74) mmol/L in the first, 2.36 (±0.89) mmol/L in the second, 1.91 (±0.80) mmol/L in the third strata, and 2.51 (±0.80) mmol/L in the fourth strata, respectively. The rate of patients exhibiting an LDL-C <1.4 mmol/L (55 mg/dl) was 9% at admission, 15.7% at discharge (*p* < 0.001 as compared to admission), 15.6% at the 3month follow-up, and 15.1% at the 12-month follow-up, respectively (Figure 5). Only few patients had LDL-C-levels below 40 mg/dl (<1 mmol) at baseline (*n* = 18), discharge (*n* = 8), 3-months-follow-up (*n* = 12), and 12-months-follow-up (*n* = 9), respectively.

## Discussion

Cardiac rehabilitation is an essential part of tertiary prevention for the long-term success of CHD treatment (8–12). Previous reviews of 148 randomized trials, which involved more than 98,000 patients, have shown that CR reduces both cardiovascular and total mortality in patients with CHD (13, 14). However, a recent review showed not only a reduction in cardiovascular mortality, but also an improvement in quality of life (15), whereas another recent review showed a reduction in all-cause mortality (8).

In Germany, a multimodal CR for 3 weeks at specialized rehabilitation clinics represents the standard of care in most patients with CHD after ACS or CABG (10, 11). Indeed, 50%–70% of patients were treated by CR after STEMI, NSTEMI, or CABG surgery, which has also been shown to improve outcome in patients with CHD (10, 16–18). As part of such multimodal rehabilitation drug therapy was optimized and patients were educated about drug effects (and side effects), which led to a better compliance of drug treatment (10, 16–18).

In the present multicenter registry study, 1,100 patients with CHD at six rehabilitation clinics in Germany were included, which presents a picture of the current treatment situation during and after CR.

The recruited patients were typical for a patient cohort with CHD in CR as shown by a mean age of 64 years, more than 75% males, and more than one third of patients with diabetes (10, 16–18). It is also typical for multimodal rehabilitation that patients receive an intensive rehabilitation program including exercise, psychosocial support, training for healthier nutrition (data not presented), and other treatments, which has been reported previously in other studies as well (8). Such a program results in reduction of waist circumference (but not BMI), systolic blood pressure, and heart rate when comparing the start and end of the rehabilitation period. This positive effect on vital parameters was also seen during the 12-month follow-up (see Table 2) and has also been demonstrated previously in other studies (16, 17).

### LLT

Drug therapy was adjusted during the rehabilitation period in the current study; in particular, LLT was optimized (Table 1, Figures 1–3), which led to a significant reduction in LDL-C levels during CR (Figures 4, 5). This was achieved by switching from low-potency simvastatin to high-potency atorvastatin, with only a small percentage of other statins being used. In addition, the significant reduction in LDL-C was achieved by increasing the proportion of patients treated with ezetimibe. In contrast, PCSK9 inhibitors were only given to a small number of patients during and after CR, since the initiation of expensive treatments such as PCSK9-inhibitors are not reimbursed in cardiac rehabilitation in Germany by the German healthcare systems. Thus, PCSK9-inhibitors are not often prescribed during cardiac rehabilitation. Comparable results regarding the optimization of statins during CR, which led to an increase in patients in the treatment targets of LDL-C, were published in 2019 (11). After 12 months, the number of patients on statin therapy (94%) was higher than expected in our study, which is not in line with other current publications. In a recent trial from Germany, 72.9% of hospitalized patients with CHD were still on statin therapy after 12 months (19). In a study from the US including almost 3 million patients with CHD, the rate of patients still on statin therapy 1 year after the index event was 79% (20). In the EUROASPIRE IV survey, 90.4% of CHD patients were on statin therapy at discharge from hospital, decreasing to 86% at 12 months (21). Recent results from the EUROASPIRE V registry have shown 80% adherence to statin therapy in patients with CHD events or interventions after 6 months (22). Other studies suggest that in real life between 33% and 75% of patients with cardiovascular disease discontinue their statin therapy within 1 year after initiation (23, 24), which has been also shown in patients with type 2-diabetes (25). In Germany, high non-persistence rates were shown regarding LLT, with the lowest persistence rates observed with statin in a recent trial including approx. 900,000 patients (26). Indeed, nonadherent patients with CHD typically discontinue new statin medications after filling only one prescription (27). Further studies showed that low adherence to statins negatively impacts clinical outcomes (23). Here, multimodal CR is effective as previously shown in studies such as the OMEGA trial, which reported an improved adherence to statin therapy after 12 months in patients after myocardial infarction and CR (81.9% without CR vs. 89.8% with CR) (17).

Although a high number of patients in the present study adhered to statin therapy after 12 months, the dosage of statins was reduced, and the proportion of patients receiving low-potency simvastatin increased, whereas the proportion of high-potency atorvastatin decreased during the 12-month follow-up period (Figures 2, 3). Such a trend towards changing low-potency to high-potency statins has been shown in previous studies but adherence to statin therapy in our post-CR patient group after 12 months was much higher than reported in the published data (94.1% in our group compared to 73%–86% in other groups in “real life”) (19–21). This positive effect might be related to participation in CR. Surprisingly, approximately ¼ of patients were still on ezetimibe after 12 months, a drug which was mostly initiated during the CR period in the current study. This positive development may change the attitudes of general practitioners regarding the significance of LLT in treating patients. However, the physician perspective has a major role and a more appropriate education for physicians should be considered to increase the achievement of LDL-C targets also within a rehabilitation program (28, 29).

### Treatment targets

In the current guidelines from the European Society of Cardiology (ESC) patients at very high risk, such as patients with ASCVD, should receive LLT to reach a treatment target of LDL-C below 1.4 mmol/L (55 mg/dl) and a reduction by 50%, independent of initial LDL-C values (2). These recommendations derive from statin and ezetimibe therapy and recent trials on PCSK9 inhibitor therapy in combination with high-dose statins, which showed clearly that the lowest LDL-C is the best LDL-C in high-risk patients (2, 5, 6). Data presented here show a significant decrease of 13.2% in median LDL-C values, more precise an absolute decrease of 2.27 mmol/L (1.80/2.84, 88.4 mg/dl) at admission to 1.97 mmol/L (1.57/2.47, 76.7 mg/dl) at discharge (*p* < 0.001 as compared to admission) was observed. Results of the PATIENT CARE registry of 1,408 patients in Germany after myocardial infarction showed comparable results, the LDL-C decreasing from the initial value of 2.49 (97 mg/dl) ± 0.83 mmol/L to 2.01 (78.3 mg/dl) ± 0.66 mmol/L during CR (11). However, our data also show that the proportion of CHD patients in the new target range below 1.4 mmol/L (55 mg/dl) was small (15.8% at the end of the rehabilitation to 15.1% at the end of the follow-up period, Figure 5) despite the fact that a high proportion of patients – more than 94% – were on statin therapy and 25% on ezetimibe therapy. With the presentation of the current treatment situation in this study during, and especially after CR with LDL-C values of 1.94 mmol/L (75.5 mg/dl) at 3 months and 1.94 mmol/L (75.5 mg/dl) at 12 months, we could show that the lipid-lowering effect associated with CR had stabilized over the follow-up period of 12 months.

### Subgroup analyses

#### Gender

Fewer women than men have been enrolled in statin trials, and *women achieve guideline-recommended LDL-C levels less often than men* (30, 31). Wether LLT and especially statin treatment induced changes in lipids were similar in women and men, has been controversial (30). E.g., a meta-analysis including 174,000 patients showed that statins are similarly efficient in reducing LDL-C levels (and the risk of major adverse cardiovascular events) in both sexes (31, 32). In the present study sex differences cannnot be observed regarding LLT (Figures 1B,C). However, although LDL-C was reduced over time both in males and females in the presented data, the effect was only significant in male patients (0.35 mmol/L *p* < 0.001 over time vs. female patients 0.21 mmol/L *p* = 0.11, Figure 4).

#### Age

Although older people are underrepresented in randomized LLT trials, and the use of LLT declines with increasing age, the 2019 ESC/EAS Guidelines for the management of dyslipidaemias stated *the available evidence from trials indicates that statin therapy produces significant reductions in major vascular events irrespective of age* (2). This recommendation was supported by a recent analysis from the FAST-MI (French Registry of Acute ST-Elevation or Non-ST-Elevation Myocardial Infarction) program. Here, a high intensity LLT was asscociated with reduced mortality also in patients with an age above 85 years (33). In the present study age differences cannnot be observed regarding LLT (Figures 1D,E). However, although LDL-C was significantly reduced over time in middle-aged patients (50–70 years, *p* < 0.001) and elderly patients (>65 years, *p* < 0. 001), respectively, the reduction in LDL-C was less pronounced in patients with an age <50 years (*p* = 0.11). This may be related to low number of younger patients in our analysis (*n* = 107, Figures 4D,E).

### Limitations

Limitations of this study include the observational, nonrandomized design and the nature of the registry data source. The data on medications and doses were obtained from patients but the adherence to the prescribed therapy were not generally checked or queried by professionals. Moreover, this was an observational study without a control group to compare the results of CR with those of patients with CHD post-ACS or CABG who did not participate in CR. Therefore, any data on effects of CR must be interpreted with caution.

However, it is difficult to establish a study design with a control group in Germany owing to social laws (after ACS or CABG every patient has the right to receive CR). To bring the data quality to a high level, the Coordination Center for Clinical Studies, Martin Luther-University Halle Wittenberg, Germany (KKS Halle) performed monitoring of the study, and patients were consecutively enrolled on a prospective basis. For clinical reasons, the treating physicians in CR may assign patients to different drugs based on disease severity, disease duration, presence of comorbidities, and other factors. This can potentially introduce allocation or channeling bias and confound the association between treatment and outcomes. On the other hand, this study represents the everyday management of CHD patients in CR in Germany. As only few inclusion or explicit exclusion criteria were applied, patients typically eligible for the CR population were documented (including those with comorbidities and concomitant medication). Notably, in nonparticipating centers and for nonparticipating patients the situation may be different, as those willing to participate may be more adherent to guideline-oriented therapy than those declining participation in prospective studies or CR in general. In general, the healthcare system in Germany achieves high levels of guideline mandated care, explaining the relatively high uptake of statin and ezetimibe in the studied population. Such high rates of uptake may limit the generalizability of the dataset from the studied population to other population groups with lower (or higher) levels of implementation of guideline mandated care.

Since other statins does not make any relevant proportion of the prescribed statins in this study simvastatin was interpreted equivalent to low intensity and atorvastatin to high intensity statin therapy (e.g., in Figures 2,3). This differs from the common classification in low, moderate, or high intensity statin therapy. Furthermore, since we do not have the baseline values of our patients (96.1% were already on statins, and 8.9% on ezetemibe on study entry) we cannot calculate how often the alternative 50% goal of LDL-C under LLT-therapy treatment was achieved in our patients.

### Conclusion

Compared to published data, a high proportion of patients was on lipid-lowering therapy with statins and ezetimibe during and after CR in this study, which may be associated with participation in CR. Despite this good treatment, only a minority of patients achieved the current recommendation of LDL-C below 1.4 mmol/L (55 mg/dl). With PCSK9-inhibitor treatment in addition to a high-dose statin therapy and ezetimibe, a higher proportion of patients might reach treatment targets. This therapy, however, was only used in a few patients in this registry study.

## Data Availability

The raw data supporting the conclusions of this article will be made available by the authors, without undue reservation.
