# Supplementary figures and images for: Lipid-lowering therapy (LLT) in 1,100 cardiac rehabilitation patients with coronary heart disease: the LLT-R(ehabilitation) registry
Source: Front Cardiovasc Med. 2025 Apr 24;12:1549935. doi: 10.3389/fcvm.2025.1549935 (PMC12059688; doi:10.3389/fcvm.2025.1549935)

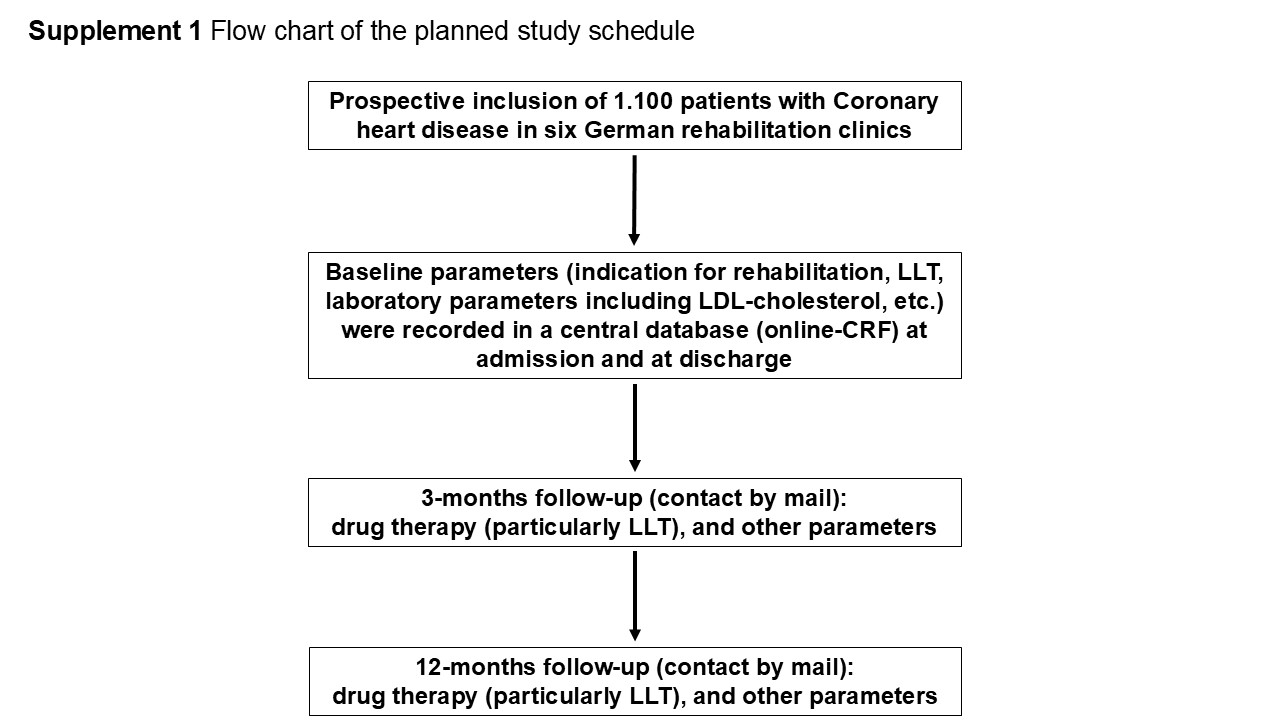

Supplement: Supplementary file 1 [file Image1.jpeg]
